# Development of Metal Plate with Internal Structure Utilizing the Metal Injection Molding (MIM) Process

**DOI:** 10.3390/ma6125878

**Published:** 2013-12-12

**Authors:** Kwangho Shin, Youngmoo Heo, Hyungpil Park, Sungho Chang, Byungohk Rhee

**Affiliations:** 1 Korea Institute of Industrial Technology, 7-46 Songdo-dong Yeonsu-gu, Incheon 406-840, Korea; E-Mails: ppori@kitech.re.kr (K.S.); php76@kitech.re.kr (H.P.); 2 Gaon Solutec, 255 Suwonchon-ro Paldal-gu, Suwon City 442-010, Korea; E-Mail: csh6336@naver.com; 3 Department of Mechanical Engineering, Ajou University, San 5, Woncheon-dong, Yeongtong-gu, Suwon, Kyungki-do 443-749, Korea; E-Mail: rhex@ajou.ac.kr

**Keywords:** viscosity, stainless steel powder feedstock, metal injection molding, flow characteristics, internal structure, sacrificed polymer insert, sintering

## Abstract

In this study, we focus on making a double-sided metal plate with an internal structure, such as honeycomb. The stainless steel powder was used in the metal injection molding (MIM) process. The preliminary studies were carried out for the measurement of the viscosity of the stainless steel feedstock and for the prediction of the filling behavior through Computer Aided Engineering (CAE) simulation. PE (high density polyethylene (HDPE) and low density polyethylene (LDPE)) and polypropylene (PP) resins were used to make the sacrificed insert with a honeycomb structure using a plastic injection molding process. Additionally, these sacrificed insert parts were inserted in the metal injection mold, and the metal injection molding process was carried out to build a green part with rectangular shape. Subsequently, debinding and sintering processes were adopted to remove the sacrificed polymer insert. The insert had a suitable rigidity that was able to endure the filling pressure. The core shift analysis was conducted to predict the deformation of the insert part. The 17-4PH feedstock with a low melting temperature was applied. The glass transition temperature of the sacrificed polymer insert would be of a high grade, and this insert should be maintained during the MIM process. Through these processes, a square metal plate with a honeycomb structure was made.

## 1. Introduction

Recently, structural products operating under a high pressure and load environment have been developed using metal. For functional requirements, the products are created with special shapes and structures. A metal is utilized frequently due to its strength, stability, formability and cost. The honeycomb structure is a well-known and useful structure able to endure severe compression load conditions [1]. These types of plates were used for insulating panels, pressure vessel chassis and floor panel *etc*. When a structural plate with a honeycomb shape is made with metal, there are several necessary parts, such as the upper plate, lower plate and inner parts. Furthermore, there are several important blanking tools and bending dies meant for manufacturing metal plates and internal structure parts. There are also secondary processes, such as welding, joining (e.g., bolting, riveting, *etc*.) and bonding methods, to accomplish a honeycomb structure. These schemes, which need other facilities, may be time-consuming and expensive. If the size of a metal plate is small, the secondary processes will be more difficult to implement, due to size effects.

**Figure 1:**
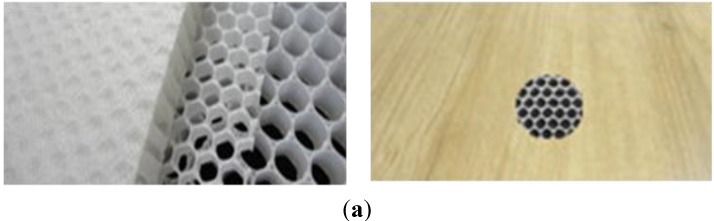

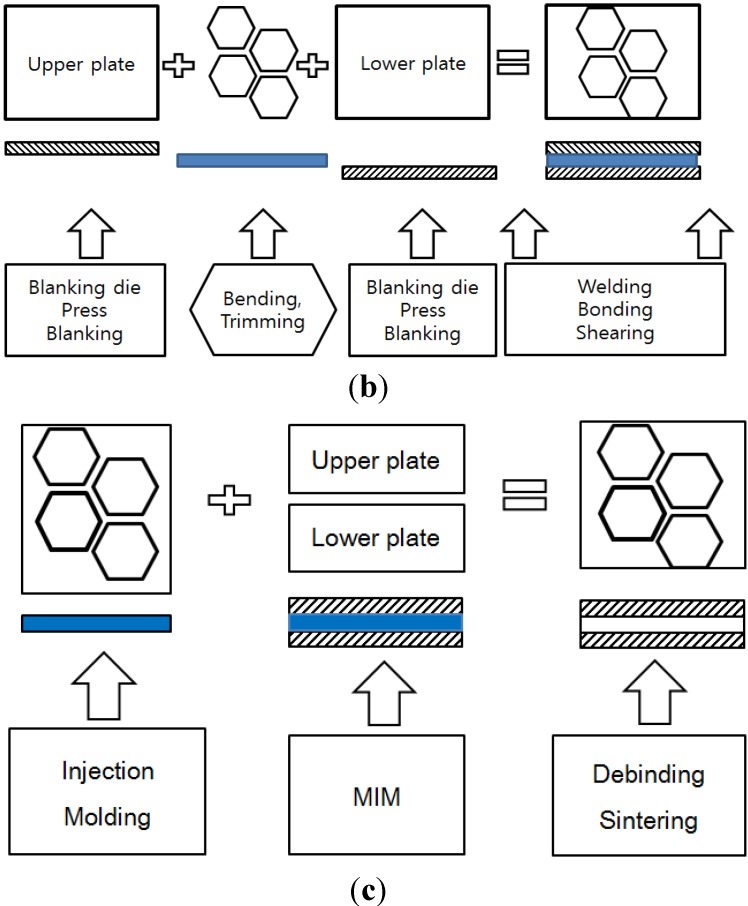
Schematic diagram for the manufacturing of a double-sided metal plate with an internal structure: (**a**) examples parts with an internal structure; (**b**) traditional method; (**c**) suggested metal injection molding (MIM) method.

The metal injection molding (MIM) process could be used to fabricate complicated metal products using metal powder and sequential debinding and sintering processes [2]. Figure 1 shows the examples, the schematic diagram comparing the traditional blanking and joining processes working as the MIM process. In consideration of the metal injection molding process, two types of injection molds were prepared in this study: one was plastic injection molds to make the sacrificed polymeric insert part, and the other was metal injection molds. Through a preliminary design study, the final size of the sacrificed polymer part was set to 22 mm × 22 mm × 0.8 mm thickness, while the green part was rendered smaller than the polymer insert at 20 mm × 20 mm × 2.4 mm thickness to fix the sacrificed insert to a suitable position of the cavity area in the metal injection mold. The total thickness was designed as the sum of the upper plate, lower plate and sacrificed part. For the prevention of the metal segment’s warpage, the thickness of the upper and lower plate was set to 0.8 mm each. The size was checked after a sintering process. To decide the suitable material for the sacrificed polymer insert, computer simulations were implemented.

## 2. Measurements of Viscosity for SUS 316 L Feedstocks

Generally, when the feedstocks are compared with polymers, these have a higher thermal conductivity that leads to fast solidification and a higher viscosity [3]. For this reason, high injection pressure appeared at the entrance of the cavity for the filling of feedstocks. Additionally, a high pressure drop occurred until the feedstock passed through the gate with a narrow cross section. This behavior would affect molding defects, such as short shot, nonhomogeneous shrinkage, binder separation, *etc*. These also affect the deformation of the internal insert part during the filling phase of metal injection molding. Thus, CAE (Computer Aided Engineering) analysis was required to predict the complex flow behavior of feedstocks for manufacturing a suitable part.

**Figure 2:**
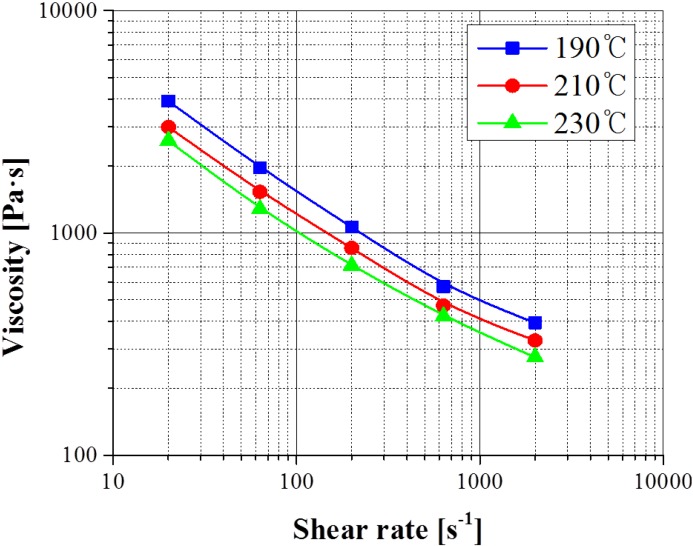
Result of the measured viscosity of 316 L feedstocks.

In this study, the measurement of viscosity was carried out to analyze the flow behavior of stainless steel powder 316 L using CAE analysis. The Catamould 316 L material made by BASF Corporation was used. The stainless steel powder volume loading is about 62% [4,5]. A Capillary Rheometer (Goettfert Rheo-Tester 1000) was used to measure the viscosity for 316 L feedstocks. Capillary tubes with a diameter of 1.0 mm and with lengths of 10, 20 and 30 mm were employed. The measured viscosity applied both Bagley’s correction to correct the pressure drop of the entrance and exit effects and Rabinowitsch correction to correct the non-linearity of the shear rate. The shear rates applied ranged from 25 (s^-1^) to 2,600 (s^-1^) considering the wall slip and the binder separation of the feedstock. Figure 2 shows the measured viscosity for 316 L feedstocks at 190, 210 and 230 °C. The result of measured viscosity was higher than for conventional thermoplastic materials, because 316 L feedstocks consisted of stainless powder and wax-based polymer. The shear thinning by increasing the shear rates was also raised higher than plastic, due to the effects involving the 316 L metal powder [6,7,8,9].

## 3. Fabrication of Metal Plate with Internal Honeycomb Structure

**Figure 3:**
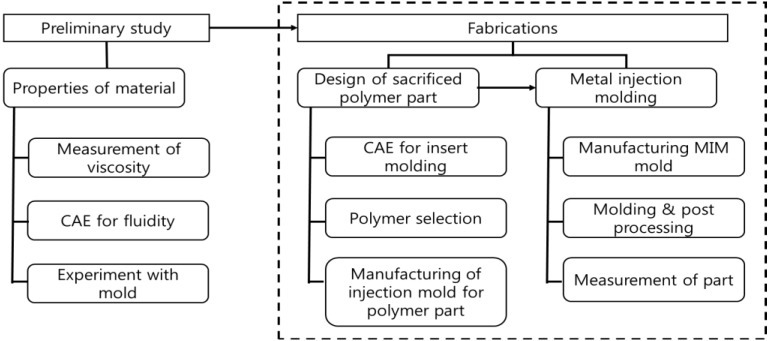
Fabrication steps for metal plate with internal structure (square zone). CAE, Computer Aided Engineering.

Fabrications of metal plates with an internal structure using the MIM process implemented several steps, as shown in Figure 3.

### 3.1. Design of Sacrificed Polymer Insert with Structure

For the manufacturing of double-sided metal plates with internal structure, a sacrificed part and polymer insert were necessary [10,11]. The initial design of the structure of the final part was executed as a square type of 30 mm with a total thickness of 2.4 mm, including a polymer insert of 0.8 mm and double-sided metal plates of 0.8 mm each. The internal structure was designed as a honeycomb structure. Each cell structure was connected to ensure the material removal function at the debinding and sintering stages. This connection area also played a role in filling and fixing.

In order to check the feasibility of the sacrificed part in the metal injection molding process, the pre-injection molding was implemented with the transparent polymer resin employing sacrificed polymer parts as inserts. After that, the final parts assembled were examined with the naked eye. In this study, three types of polymer resins, such as low density polyethylene (LDPE), high density polyethylene (HDPE) and polypropylene (PP), which have low viscosities and easy debinding properties, were utilized. There was no problem in filling the sacrificed polymer insert.

**Figure 4:**
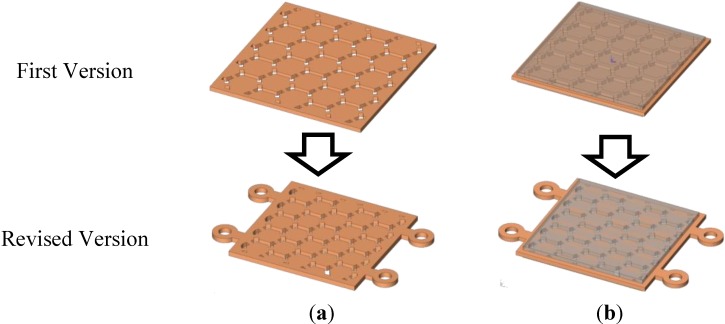
Modification for the metal plate with an internal structure (honeycomb): (**a**) sacrificed polymer insert; and (**b**) sacrificed insert with metal plate.

Some problems occurred for the filling of the melt, due to the operation of the sacrificed polymer part as an obstacle. The sacrificed polymer insert influenced the obstacles for the feedstock flow, and it was deformed and moved in the cavity. The upper and lower cavities for feedstock filling did not meet any obstacles, and those were filled faster than the insert region. This was influenced by the unbalanced injection pressure and caused the deformation of the polymer insert. The insert was fixed by a mold plate only, and the fixing mechanism was not stable at the center of the cavity during the metal injection process. Figure 4 shows the shape and structure of the target part. The modifications of the insert were necessary for the size, the shape of the sacrificed part and the fixing function to prevent the moving of the insert in the cavity of the MIM mold [12].

**Figure 5:**
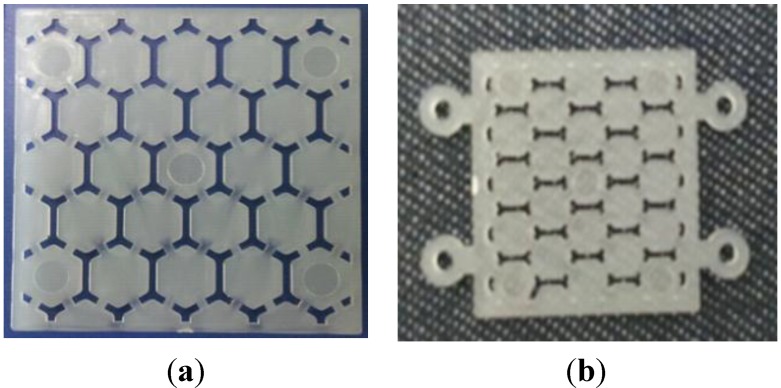
The molding of the sacrificed polymer parts: (**a**) first polymer insert molding; and (**b**) revised polymer insert molding.

For a successful MIM process, the size was reduced to 22 mm × 22 mm × 0.8 mm, and four-hole structures were added to prevent movement within the cavity during the metal injection molding process. Figure 4 shows the modification of the sacrificed polymer insert part. Figure 5 shows the first version of the sacrificed polymer insert molding and the revised version. The injection molds were designed, and two cavity types were made for balancing of the flow and prevention of deformation under injection pressure [13].

### 3.2. CAE Simulation for the Checking of Filling during the MIM Process

#### 3.2.1. Selection of Material for Sacrificed Polymer Insert and Core Shift Analysis

The sacrificed polymer insert was affected by injection pressure during the metal injection molding process. If the pressure difference between the upper and lower plate occurred during the metal injection molding process, it would affect the movement and deformation of the polymer insert and also change the thickness of the cavity [14,15]. The insert had a suitable rigidity to endure the filling pressure. The core shift analysis using commercial simulation software (Autodesk Moldflow Insight 2012TM) was conducted to predict the deformation of the insert part [16]. Table 1 shows the mechanical properties of the polymer used for analysis. Table 2 shows boundary conditions for analysis.

**Table 1:** Mechanical properties of polymers. PP, polypropylene; HDPE, high density polyethylene; LDPE, low density polyethylene.

Material Type	Unit	PP Honam A-372	PP Sabic 513MNK10	HDPE Sabic CCX912	LDPE Sabic 1965T
Elastic Modulus	MPa	3,046	1,340	911	124
Poisson’s Ratio	–	0.425	0.392	0.426	0.41
Shear Modulus	MPa	720	481.3	319.4	43.97
Thermal Expansion Coefficient	°C^−1^	6.79 × 10^5^	9.05 × 10^5^	1.50 × 10^4^	1.80 × 10^4^
Glass Transition Temperature	°C	135	123	114	90

**Table 2:** Boundary conditions for CAE simulation.

Process parameter	Unit	Value
Melt Temperature	°C	170
Mold Temperature	°C	120
Injection Time	s	0.8
Holding Pressure *	%	80
Holding Time	s	1.5
Cooling Time	s	20

* Percentage value is based on the maximum injection pressure.

**Figure 6:**
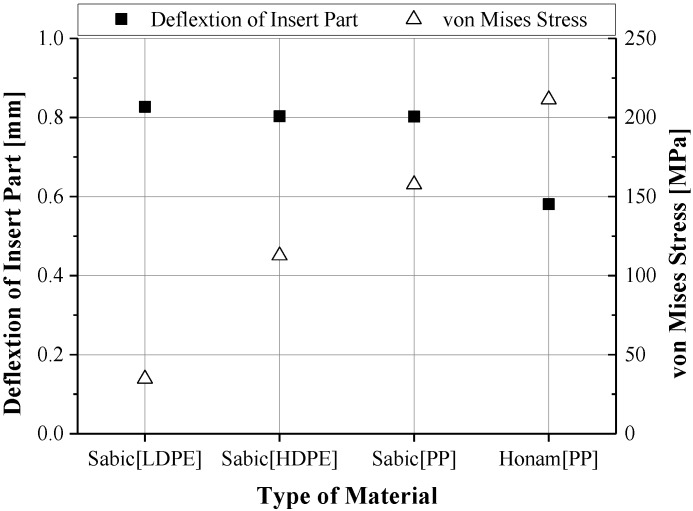
The deformation of the insert and von Mises stress according to the types of materials.

**Figure 7:**
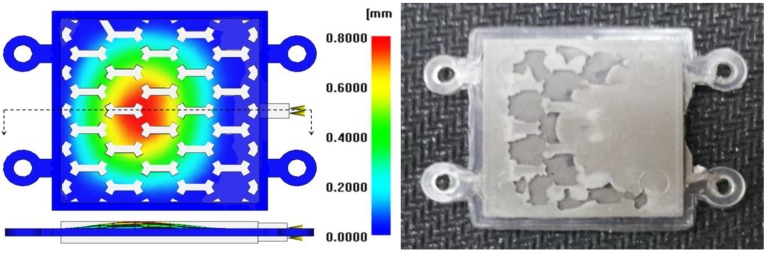
The deformation result from core shift analysis and a photo of the experimental green part.

The sacrificed polymer inserts with a low elastic modulus, such as PP, HDPE and LDPE, exhibited large warpages, as shown in Figure 6. The Honam PP material with a high elastic modulus showed the least deformation and high von Mises stress. For this reason, the Honam PP material (A-372) with high rigidity compared with the others was selected. In CAE analysis, the deformation of the sacrificed polymer insert was reduced by about 50%. Figure 7 shows the deformation in the simulation result and the photo of the experimental green part for the LDPE polymer inserts with a low elastic modulus.

#### 3.2.2. Metal Injection Molding Condition

It is preferable to use low-injection velocity for the prevention of binder segregation during the metal injection filling phase. High injection velocity is necessary to cope with the increase of injection pressure caused by the rapid cool down of molten feedstocks, which have metal granules with high thermal conductivity aimed at filling the cavity [3,17,18]. To find an adequate injection condition, metal injection molding and structural simulations were conducted. CAE simulations were executed according to the variations of injection times from 0.4 s to 2.0 s with 0.2 s steps. Those were focused on maximum shear stress, injection pressure, deformation of the polymer insert and distribution of von Mises stress [19,20,21].

**Figure 8:**
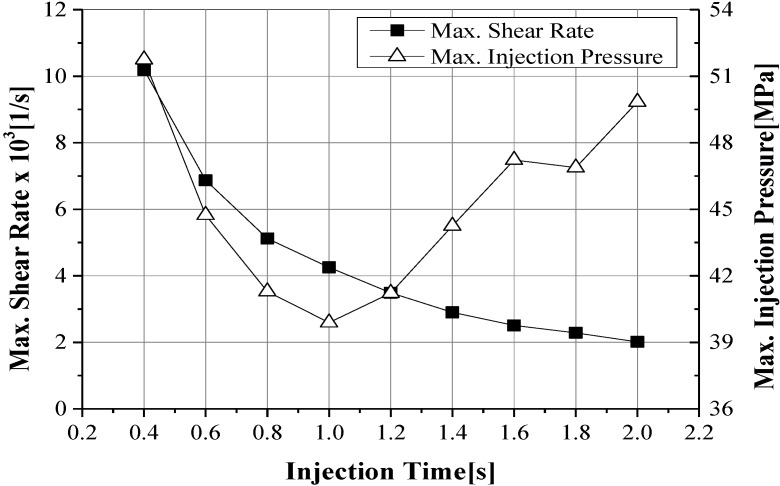
Maximum shear stress and injection pressure according to injection time.

**Figure 9:**
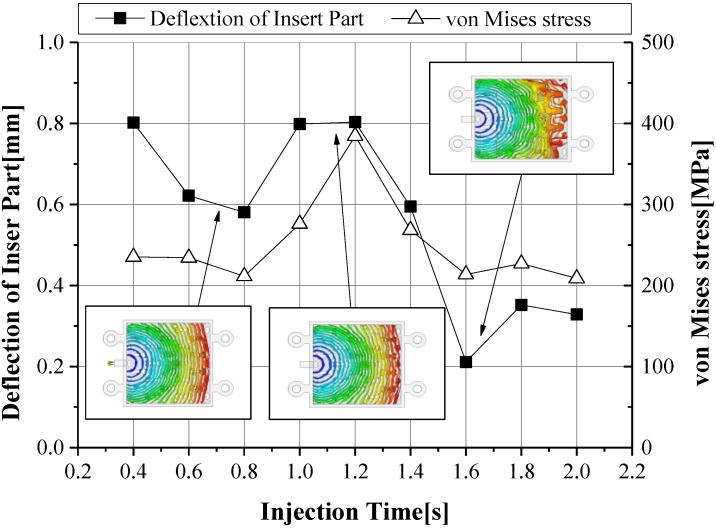
Deformation of the insert and von Mises stress according to injection time.

**Figure 10:**
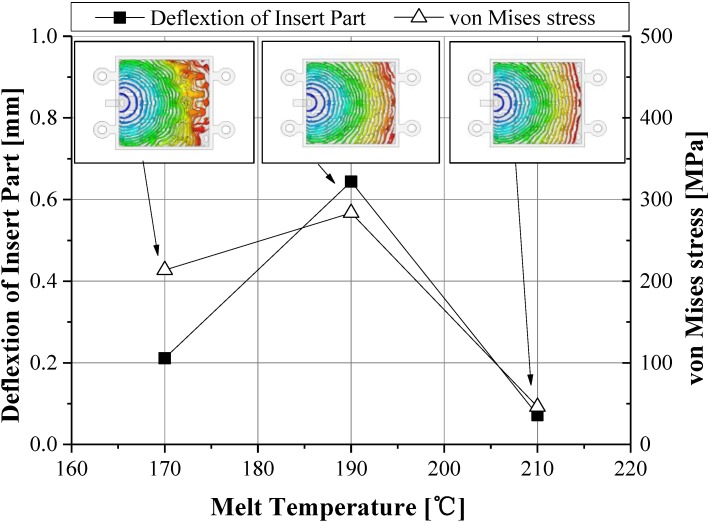
Deformation of insert and von Mises stress according to melt temperature.

**Figure 11:**
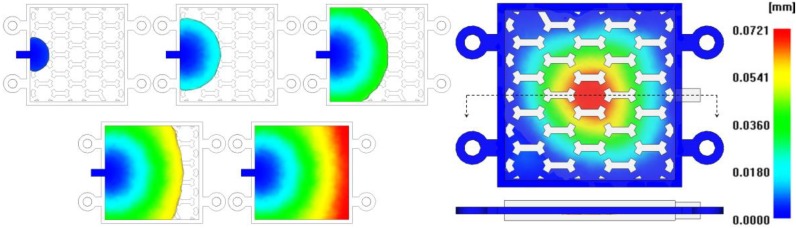
Filling pattern and the distribution of deformation.

The maximum shear stress decreased according to the increase of injection time and injection pressure, showing a U shape tendency on a 1.0 s basis, as illustrated in Figure 8 [22,23]. For consideration of the deformation results of the insert, a minimum deformation and minimum von Mises stress were shown at an injection time of 1.6 s, as shown in Figure 9. At this injection time, the deformation of the insert was presented at about 0.2 mm and an imbalance filling behavior occurred at the end of the filling area. This was caused by the increase of viscosity from the rapid decrease of the melt temperature. It was possible then to reduce the deformation of the insert, but it affected the inferior metal part with an air trap. In order to improve the quality of the end of the filling area, the deformation analysis of the inserts was implemented. In Figure 10, the filling quality was improved at a 190 °C melt temperature, but the amount of deformation and von Mises stress were increased. At a 210 °C melt temperature, the filling quality showed balanced filling, with the deformation occurring at about 0.07 mm, an adequate value. Figure 11 shows the filling pattern and the distribution of deformation.

**Figure 12:**
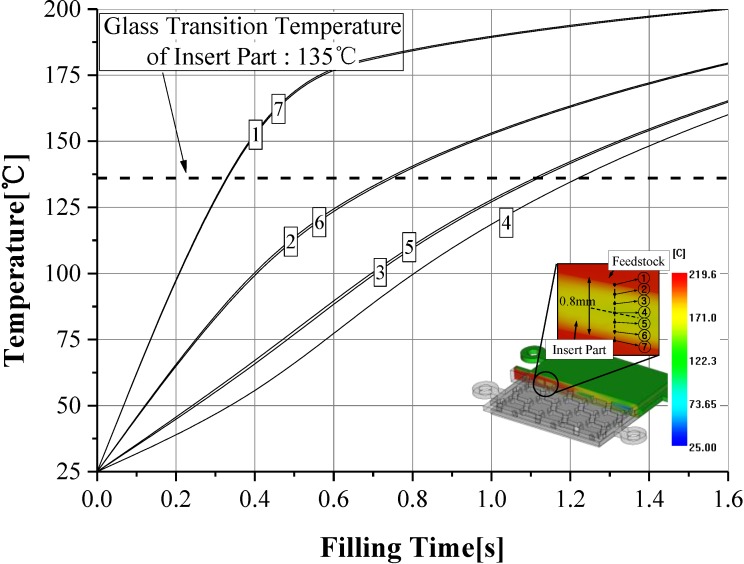
Distribution of the cross-section temperature in the sacrificed polymer insert for SUS (stainless steel) 316 L feedstocks.

However, the melting temperature of 210 °C is higher than the glass transition temperature of 135 °C with the sacrificed polymer insert. The high melting temperature of the feedstock occurred, re-melting on the surface of the sacrificed polymer insert. This was caused by reducing the geometric accuracy in the internal honeycomb structure of the final product. Figure 12 shows the cross-section temperature of the thickness direction for the sacrificed polymer insert in the case of 316 L feedstocks at a 210 °C melting temperature. The temperature of the sacrificed polymer insert was higher than the glass transition temperature of 135 °C, due to the effects the high melt temperature of 316 L feedstocks. This result means that the geometric accuracy is reduced by re-melting on the surface of the sacrificed polymer insert.

**Figure 13:**
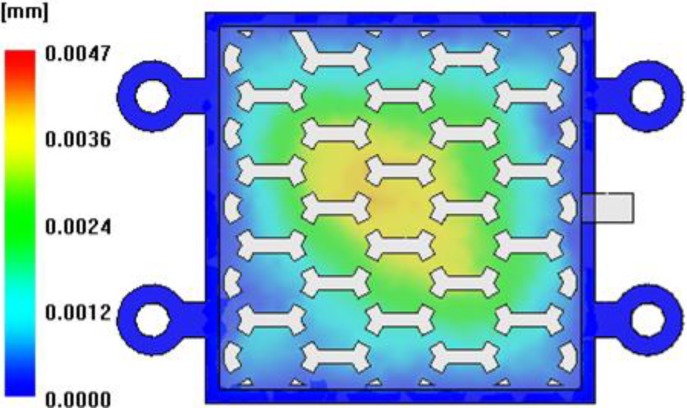
Distribution of deformation in the sacrificed polymer insert for 17-4PH feedstocks.

**Figure 14:**
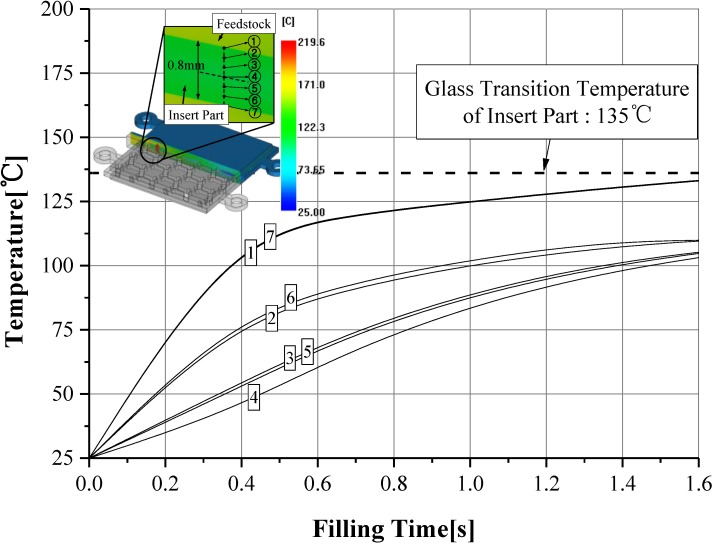
Distribution of the cross-section temperature in the sacrificed polymer insert for 17-4PH feedstocks.

In order to avoid the re-melting phenomena of the sacrificed polymer insert, 17-4PH feedstocks with a low melting temperature were applied. The viscosity model of 17-4PH feedstocks proposed by Ilica was used in this CAE analysis [24]. The melt temperature was set to 170 °C. The mold temperature was set to 60 °C. The other conditions applied process conditions of SUS (stainless steel) 316 L feedstocks. The flow patterns of 17-4PH feedstocks was similar to those of SUS 316 L feedstocks. However, as shown Figure 13, the deformation of the sacrificed polymer insert was significantly improved by about 0.004 mm. This was caused by the low viscosity of 17-4PH feedstocks. Figure 14 shows the cross-section temperature of the thickness direction for the sacrificed polymer insert in the case of 17-4PH feedstocks. The temperature of the sacrificed polymer insert was formed below the glass transition temperature. This means that geometric accuracy improves the internal honeycomb structure, because re-melting on the sacrificed polymer insert did not happen.

### 3.3. Metal Injection Molding Process and Post-Processing

The metal injection molding process was executed using stainless steel feedstocks (17-4PH 15F, solid load in a volume of 59%, D50 = 8.20 μm (−10 μm = 59.9%, 20~10 μm = 30.4%, 30~20 μm = 7.6%, +30 μm = 2.2%), wax-based binder system with stearic acid 2% and compounded at 160 °C. Insert molding was implemented in the location of the sacrificed molding. Inserts were located and fixed at cavities using four pins, as shown in Figure 5b. The sizes of the outer metal plates were set to 20 mm × 20 mm × 8 mm. The side gate type was selected to fill the metal feedstock for both outer plates.

**Figure 15:**
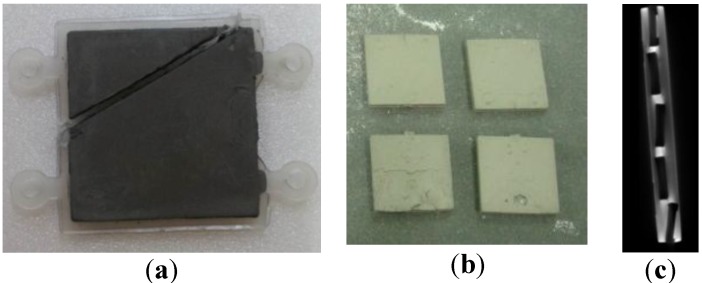
Green parts and sintered parts: (**a**) green part; (**b**) sintered part; and (**c**) section view of sintered part.

**Figure 16:**
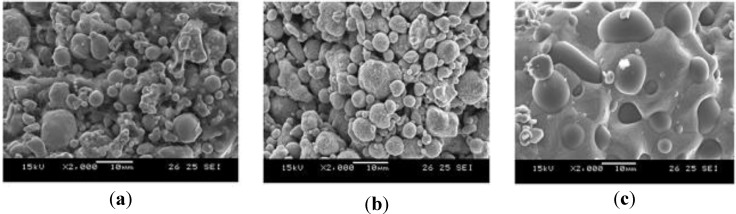
SEM images for the part at each phase. (**a**) green part; (**b**) brown part; and (**c**) sintered part.

Figure 15 shows the MIM processes and the cutting of the green part to check the filling phenomenon. The position of the gate was decided and made between the shorter sides of the fixing points. To obtain the target part, a debinding process and sintering process were adopted [25]. Debinding conditions served to increase the temperature up to 50 °C (increasing speed: 2 °C/min) and were maintained at 2 h in a normal-hexane bath. Sintering process conditions increased up to 1050 °C (increasing rate: 1.5 °C/min), were maintained for 1 h at 1050 °C and posed a decreased temperature until 800 °C (cooling rate: 5 °C/min); the power was turned off, and cooling occurred in a furnace. Figure 15 shows the green part and the sintered part, while Figure 16 shows SEM images for the green part, brown part and sintered part.

**Figure 17:**
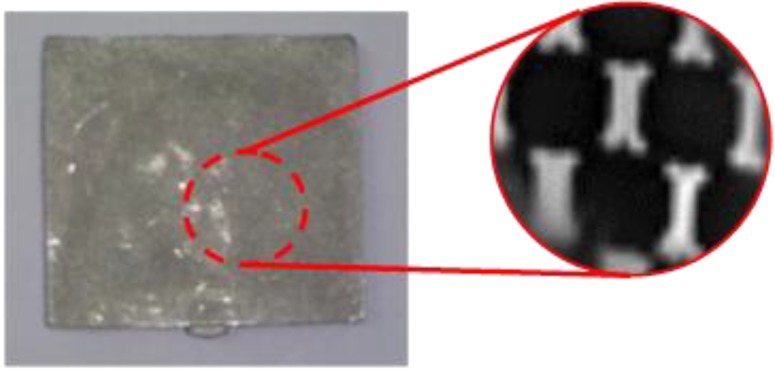
Non-destructive test (NDT) inspection view of the sintered part for checking the honeycomb structure.

To check the internal structures, an NDT (non-destructive test) was executed by X-ray, and Figure 17 shows the sintered part and internal structure [26]. It was shown that the internal honeycomb structures were well-fabricated. The thicknesses and widths were measured, and the shrinkage was checked from the green part to the sintering part. The shrinkage was about 16.3% in thickness and 15.5% in width direction.

## 4. Conclusions

In the development of a metal plate with internal structure, the following points were conducted and drawn:

(1) A stainless steel metal plate with an internal structure was developed using the recommended metal injection molding process.
(2) The sacrificed polymer insert was designed to fix and prevent movement in the MIM mold cavity, and each cell or channel was connected so as to be removed without air trapping during the debinding and sintering processes. These connections would act as channels for fluid applications.
(3) The glass transition temperature of the sacrificed polymer insert would be of high-grade, and this insert should be maintained during the MIM process.
(4) CAE simulations to find a suitable sacrificed polymer insert and an adequate metal injection molding condition were recommended to reduce the development time.
(5) The shrinkage of the target part during post-processing was about 16.3% in thickness and 15.5% in width direction. The total thickness was approximately 2.0 mm.
